# Supplementary material for: Using a continuum model to decipher the mechanics of embryonic tissue spreading from time-lapse image sequences: An approximate Bayesian computation approach
Source: PLoS One. 2019 Jun 27;14(6):e0218021. doi: 10.1371/journal.pone.0218021 (PMC6597152; doi:10.1371/journal.pone.0218021)
Supplement: S1 File — Here we describe the implementation of the strain mapping method to calculate the deformation of a tissue via estimates of the x-strain εxx, y-strain εyy, xy-strain εxy, yx-strain εyx, and displacement gradient ∇u between two images in a time-lapse sequence. (PDF) [file pone.0218021.s001.pdf]

Strain mapping method discretization

Here we describe the implementation of the strain mapping method to calculate the deformation of a tissue via estimates of the x-strain ε_{xx}, y-strain ε_{yy}, xy-strain ε_{xy}, yx-strain ε_{yx}, and displacement gradient $\nabla\mathbf{u}$ between two images in a time-lapse sequence.

For images of width n pixels and height m pixels, the properties of each pixel can be represented by an entry in an $m\times n$ matrix. For each pair of images from the time-lapse sequence, let $\mathbf{X}=(\mathbf{X}_1,\mathbf{X}_2)$ be the (x,y)-coordinate positions in the first still image in pixels and $\mathbf{x}=(\mathbf{x}_1,\mathbf{x}_2)$ be the (x,y)-coordinate positions in the second still image. The top left corner of an image is the origin, and x increases from left to right and y increases from top to bottom. The entries of $\mathbf{X}$ are therefore defined by $\mathbf{X}_1(i,j)=j-1$ and $\mathbf{X}_2(i,j)=i-1$ for $i=1,2,\ldots,n$, and $j=1,2,\ldots,m$.

We mask the pair of images so that registration occurs only in the actual location of cells. We then initialize bUnwarpJ to calculate the coefficients of the cubic B-spline map β that defines the transformation $(\mathbf{X}_1, \mathbf{X}_2) \xrightarrow{\beta} (\mathbf{x}_1, \mathbf{x}_2)$ [1]. Initializing bUnwarpJ again, we apply β to $\mathbf{X}$ by converting the transformation to "raw" data, which reports the elastically-mapped position of each pixel in the first image to the "fitted" position in the second image. We obtain $\mathbf{x}$, the mapped position of each pixel from the first image to its position in the second image, in pixels. Note that pixels outside of the mask will be mapped as well, but we will remove this extraneous data after we have obtained the strains. Next, we calculate the displacement vector $\mathbf{u}$ by

$$\begin{aligned} \mathbf{u}_1(i,j) &= \mathbf{x}_1(i,j) - \mathbf{X}_1(i,j), \qquad i=1,2,...,n, \quad j=1,2,...,m, \\ \mathbf{u}_2(i,j) &= \mathbf{x}_2(i,j) - \mathbf{X}_2(i,j), \qquad i=1,2,...,n, \quad j=1,2,...,m. \end{aligned} \tag{1}$$

The engineering, or Cauchy, strain is defined as

$$\varepsilon = \frac{\Delta L}{L_0} = \frac{L-L_0}{L_0}, \tag{2}$$

where ΔL is the change in length of the tissue, L_0 is the original length, and L is the current length. The displacement vector $\mathbf{u}$ is converted into x-strain, y-strain, xy-strain, and yx-strain by

$$\begin{aligned} \varepsilon_{xx}(i,j) &= \frac{\mathbf{u}_1(i,j)-\mathbf{u}_1(i-1,j)}{\mathbf{X}_1(i,j)-\mathbf{X}_1(i-1,j)}, \qquad i=2,3,...,n, \quad j=1,2,...,m, \\ \varepsilon_{xy}(i,j) &= \frac{\mathbf{u}_1(i,j)-\mathbf{u}_1(i,j-1)}{\mathbf{X}_2(i,j)-\mathbf{X}_2(i,j-1)}, \qquad i=1,2,...,n, \quad j=2,3,...,m, \\ \varepsilon_{yx}(i,j) &= \frac{\mathbf{u}_2(i,j)-\mathbf{u}_2(i-1,j)}{\mathbf{X}_1(i,j)-\mathbf{X}_1(i-1,j)}, \qquad i=2,3,...,n, \quad j=1,2,...,m, \\ \varepsilon_{yy}(i,j) &= \frac{\mathbf{u}_2(i,j)-\mathbf{u}_2(i,j-1)}{\mathbf{X}_2(i,j)-\mathbf{X}_2(i,j-1)}, \qquad i=1,2,...,n, \quad j=2,3,...,m. \end{aligned} \tag{3}$$

Note that all of the denominators above equal 1 pixel and the shear strains $\varepsilon_{xy} = \varepsilon_{yx}$. At this point, the strains can be visualized to show where there are contractions in the tissue ($\varepsilon < 0$) and where there are dilations ($\varepsilon > 0$) [2].

Using these strain calculations, we can numerically approximate the displacement gradient at each pixel as

$$\nabla \mathbf{u}(i,j) = \begin{pmatrix} \varepsilon_{xx}(i,j) & \varepsilon_{xy}(i,j) \\ \varepsilon_{xy}(i,j) & \varepsilon_{yy}(i,j) \end{pmatrix}, \qquad i = 2,3,\ldots,n \quad j = 2,3,\ldots,m. \tag{4}$$

We found that we could limit numerical boundary effects on the registration by ensuring the tissue was at least 200 pixels from the outer boundary of the image. To ensure the registration between images was detecting movement and not noise, we chose a time interval long enough for movement to be discernible. In our case, a time interval of 25 minutes ensured that the relative change in area between image pairs was on average more than 5%, which would correspond with strain measurements above the noise between images.

References

1. Arganda-Carreras I, Sorzano COS, Marabini R, Carazo JM, Ortiz-de-Solarzano C, Kybic J. Consistent and elastic registration of histological sections using vector-spline regularization. In: Beichel RR, Sonka M, editors. Computer Vision Approaches to Medical Image Analysis, Lecture Notes in Computer Science Vol 4241. Germany: Springer-Verlag Berlin Heidelberg; 2006. pp. 85–95.
2. Kim Y, Hazar M, Vijayraghavan DS, Song J, Jackson TR, Joshi SD, et al. Mechanochemical actuators of embryonic epithelial contractility. Proc Natl Acad Sci U S A. 2014;111: 14366–14371. doi:10.1073/pnas.1405209111
