# Supplementary material for: Using a continuum model to decipher the mechanics of embryonic tissue spreading from time-lapse image sequences: An approximate Bayesian computation approach
Source: PLoS One. 2019 Jun 27;14(6):e0218021. doi: 10.1371/journal.pone.0218021 (PMC6597152; doi:10.1371/journal.pone.0218021)
Supplement: S2 File — Here we describe the derivation of the mathematical model of single layer cell migration of Arciero et al. [11]. (PDF) [file pone.0218021.s002.pdf]

Mathematical model derivation

Here we describe the derivation of the mathematical model of single layer cell migration of Arciero et al. [1].

The cell layer is represented as a 2D compressible fluid, and the variable ρ describes the tissue density as a function of position $\mathbf{x}=(x,y)$ and t. The law of conservation of mass,

$$\frac{\partial \rho}{\partial t} + \nabla \cdot (\rho \mathbf{v}) = q, \tag{1}$$

where $\mathbf{v}$ is the velocity of the cell layer, includes the growth term q which may generally depend on space $\mathbf{x}$, time t, or density ρ, and describes the net rate of change in the number of cells within the layer.

Balance of linear momentum implies

$$\rho \frac{\partial \mathbf{v}}{\partial t} + \rho (\mathbf{v} \cdot \nabla) \mathbf{v} = \mathbf{f} + \nabla \cdot \mathbf{T}, \tag{2}$$

where the tensor $\mathbf{T}$ represents the stresses within the cell layer and $\mathbf{f}$ accounts for the force of adhesion of the cell layer to the substrate. $\mathbf{f}$ is the result of the action exerted on a material element by the substrate, i.e. the negative of traction force. It is assumed that the $\mathbf{f}$ is negatively proportional to the cell layer velocity,

$$\mathbf{f} = -b\mathbf{v}, \tag{3}$$

where b is a constant of adhesion. The cell layer is assumed to behave as a compressible inviscid fluid with the constitutive equation

$$\mathbf{T} = -p(\rho)\mathbf{I}, \tag{4}$$

where p is the pressure within the cell layer. The pressure depends on the tissue density and is taken to be positive when cells are compressed and negative when cells are stretched. Assuming acceleration is negligible and substituting Eqs. 3 and 4 into Eq. 2 we obtain the equation

$$b\mathbf{v} = -p'(\rho)\nabla\rho, \tag{5}$$

which is the relation between the velocity of cells and the gradient of tissue density; it resembles Darcy's law describing the flow of fluid through a porous medium.

Substituting Eq. 5 into Eq. 1 results in the governing equation that describes the evolution of tissue density,

$$\frac{\partial \rho}{\partial t} = \frac{1}{b} \nabla \cdot \left(\rho p'(\rho) \nabla \rho \right) + q. \tag{6}$$

Given the presence of lamellipodia on the edge of the ectoderm, i.e. tissue boundary $\partial\Omega_1^t$, we assume there is a constant force per unit length F (see Table 1 and Fig 2A-B) exerted outward at the tissue boundary that is equal in magnitude to that of the force of the cells in the interior. To express this boundary condition in mathematical notation, a function describing the forces within the tissue is necessary. In Arciero et al. [1], various constitutive relations for function $p(\rho)$ were considered, but the main relation studied was

$$p(\rho) = k \ln\left(\rho / \rho_{\text{unstressed}}\right), \tag{7}$$

where $\rho_{\text{unstressed}}$ is a parameter described in Table 1, as it gave appropriate behavior at both large and small densities. Substituting the constitutive relation in Eq. 7 into Eq. 6 gives the governing equation

$$\frac{\partial \rho}{\partial t} = \frac{k}{b} \Delta \rho + q. \tag{8}$$

Notice that the Laplacian that appears in the governing equation is due to the constitutive relation chosen for the pressure and hence the governing equation should not be thought of as reaction-diffusion equation. The Laplacian does not arise from any underlying diffusion process or Brownian motion and *k/b* should not be interpreted as a diffusion constant (Arciero et al. [1]).

One boundary condition imposed on the boundary of the cell layer is that there is a constant force per unit length F outward directed against the substrate due to lamellipodia, which requires setting $p = -F$ at the boundary [1,2] in Eq. 7 and then solving for ρ, resulting in

$$\rho = \rho_{\text{unstressed}} e^{-F/k}, \qquad \text{on } \partial\Omega_1^t. \tag{9}$$

Another boundary condition imposed on the boundary of the cell layer is a Stefan condition, which describes the speed of the moving edge. This condition comes from Eq. 5 evaluated at Eq. 9 and is

$$\mathbf{v} \cdot \mathbf{n}_1 = \left(-\frac{k}{b} \frac{1}{\rho_{\text{unstressed}}} e^{F/k} \nabla \rho \right) \cdot \mathbf{n}_1, \qquad \text{on } \partial\Omega_1^t. \tag{10}$$

where $\mathbf{v}(\mathbf{x},t)$ is the velocity of the layer and $\mathbf{n}_1(\mathbf{x},t)$ is the outward unit normal to the tissue boundary $\partial\Omega_1^t$.

On the edge of the computational domain $\partial\Omega_2$, we assume that there is no flux of cells, i.e. cells are unable to move beyond this boundary, and so we have the Neumann boundary condition

$$\nabla \rho \cdot \mathbf{n}_2 = 0, \qquad \text{on } \partial\Omega_2, \tag{11}$$

where $\mathbf{n}_2(\mathbf{x},t)$ is the outward unit normal to the edge of the computational domain $\partial\Omega_2$.

By segmenting cells in confocal images of the epithelial layer of a representative animal cap explant labeled with a GFP-membrane tag, we measured the tissue density in the epithelial layer at the initial imaging time point to be 0.0047 cells/μm^2, so we take the initial condition to be

$$\rho(x,0) = 0.0047, \qquad \text{in } \Omega^0. \tag{12}$$

References

1. Arciero JC, Mi Q, Branca MF, Hackam DJ, Swigon D. Continuum model of collective cell migration in wound healing and colony expansion. Biophys J. 2011;100: 535–543. doi:10.1016/j.bpj.2010.11.083
2. Stepien TL. Collective Cell Migration in Single and Dual Cell Layers [Internet]. University of Pittsburgh. 2013. Available: http://d-scholarship.pitt.edu/18675/
