# Supplementary material for: Using a continuum model to decipher the mechanics of embryonic tissue spreading from time-lapse image sequences: An approximate Bayesian computation approach
Source: PLoS One. 2019 Jun 27;14(6):e0218021. doi: 10.1371/journal.pone.0218021 (PMC6597152; doi:10.1371/journal.pone.0218021)
Supplement: S1 Table — 10,000 parameter sets were used per explant. (PDF) [file pone.0218021.s003.pdf]

Parameter		F/k (dimensionless)		k/b ($\mu m^2/h$)		α (h^{-1})		$\rho_{unstressed}$ ($cells/\mu m^2$)	
**Explant**	**Initial Area (mm^2)**	**Lower Bound**	**Upper Bound**	**Lower Bound**	**Upper Bound**	**Lower Bound**	**Upper Bound**	**Lower Bound**	**Upper Bound**
1	0.14	0.4949	0.5060	1700	1739	0.4678	0.4793	1528	1539
2	0.23	0.5188	0.5299	1904	1946	0.4514	0.4629	1513	1524
3	0.23	0.4112	0.4223	1937	1983	0.4376	0.4491	1528	1539
4	0.24	0.4156	0.4269	2161	2209	0.4244	0.4358	1523	1535
5	0.26	0.4980	0.5091	1903	1946	0.4585	0.4699	1519	1530
6	0.30	0.4397	0.4517	2262	2311	0.4470	0.4584	1512	1524
7	0.36	0.5084	0.5195	2047	2093	0.4504	0.4618	1515	1526
8	0.45	0.5309	0.5425	2355	2402	0.4419	0.4532	1491	1502
9	0.54	0.6276	0.6391	2653	2700	0.4658	0.4772	1468	1479
10	0.60	0.6630	0.6741	2718	2764	0.4697	0.4811	1460	1471
11	0.64	0.5430	0.5549	2508	2556	0.4497	0.4610	1480	1491
12	0.68	0.6079	0.6197	2624	2672	0.4545	0.4659	1467	1478
13	0.86	0.5499	0.5621	2502	2550	0.4423	0.4538	1482	1494
14	1.12	0.4848	0.4965	2366	2415	0.4357	0.4470	1489	1500
15	1.49	0.4585	0.4704	2318	2366	0.4397	0.4510	1488	1499
16	1.73	0.5779	0.5898	2614	2663	0.4550	0.4663	1469	1481
17	2.14	0.5524	0.5649	2556	2605	0.4419	0.4532	1464	1476
18	2.57	0.7522	0.7621	2943	2987	0.5011	0.5122	1440	1451
