# Supplementary figures and images for: Using a continuum model to decipher the mechanics of embryonic tissue spreading from time-lapse image sequences: An approximate Bayesian computation approach
Source: PLoS One. 2019 Jun 27;14(6):e0218021. doi: 10.1371/journal.pone.0218021 (PMC6597152; doi:10.1371/journal.pone.0218021)

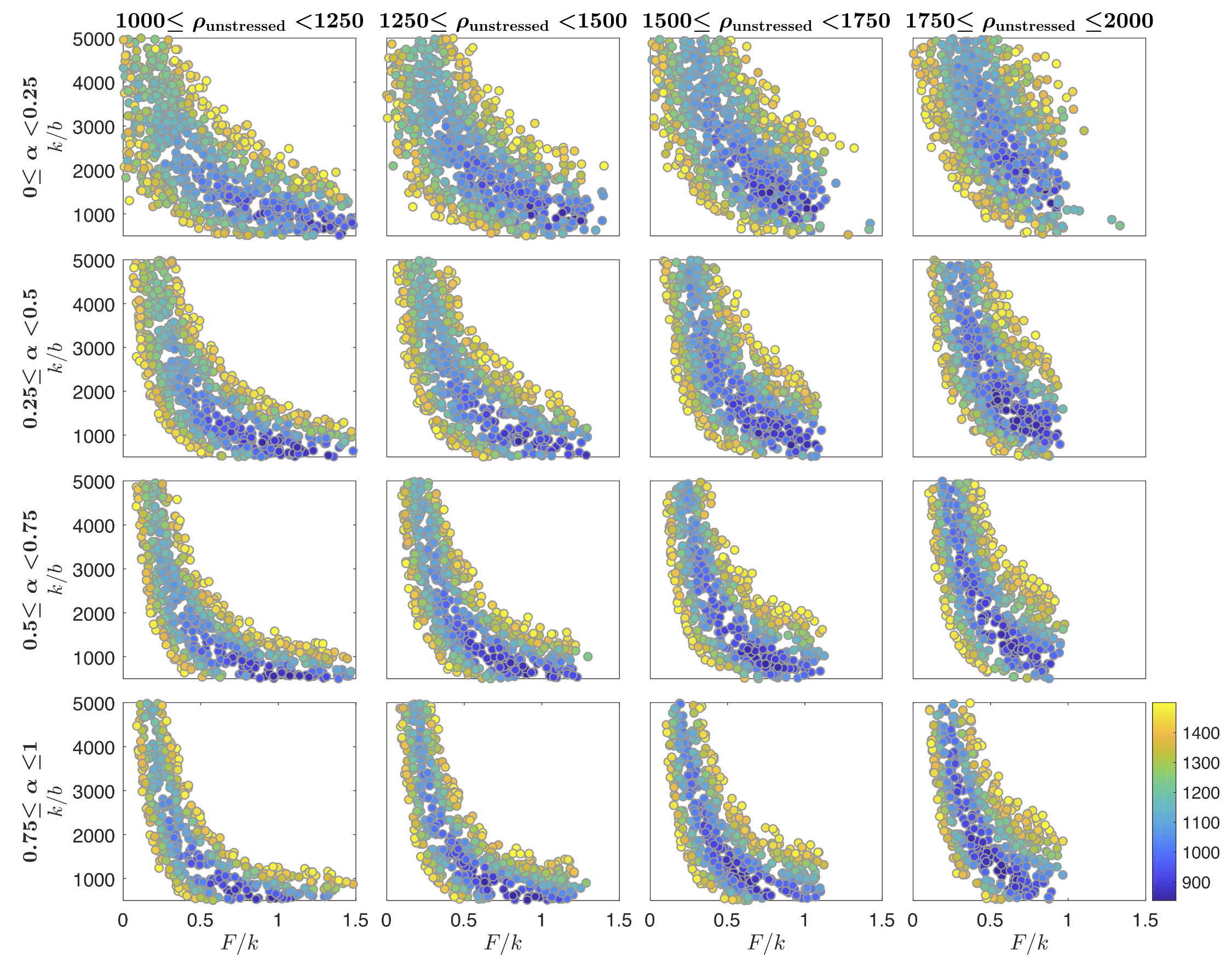

Supplement: S1 Fig — Each circle indicates an accepted parameter value set found using the approximate Bayesian computation rejection method (cf. Fig 3A), and the color of the circle corresponds with its calculated error (Eq 12). (TIFF) [file pone.0218021.s004.tiff]

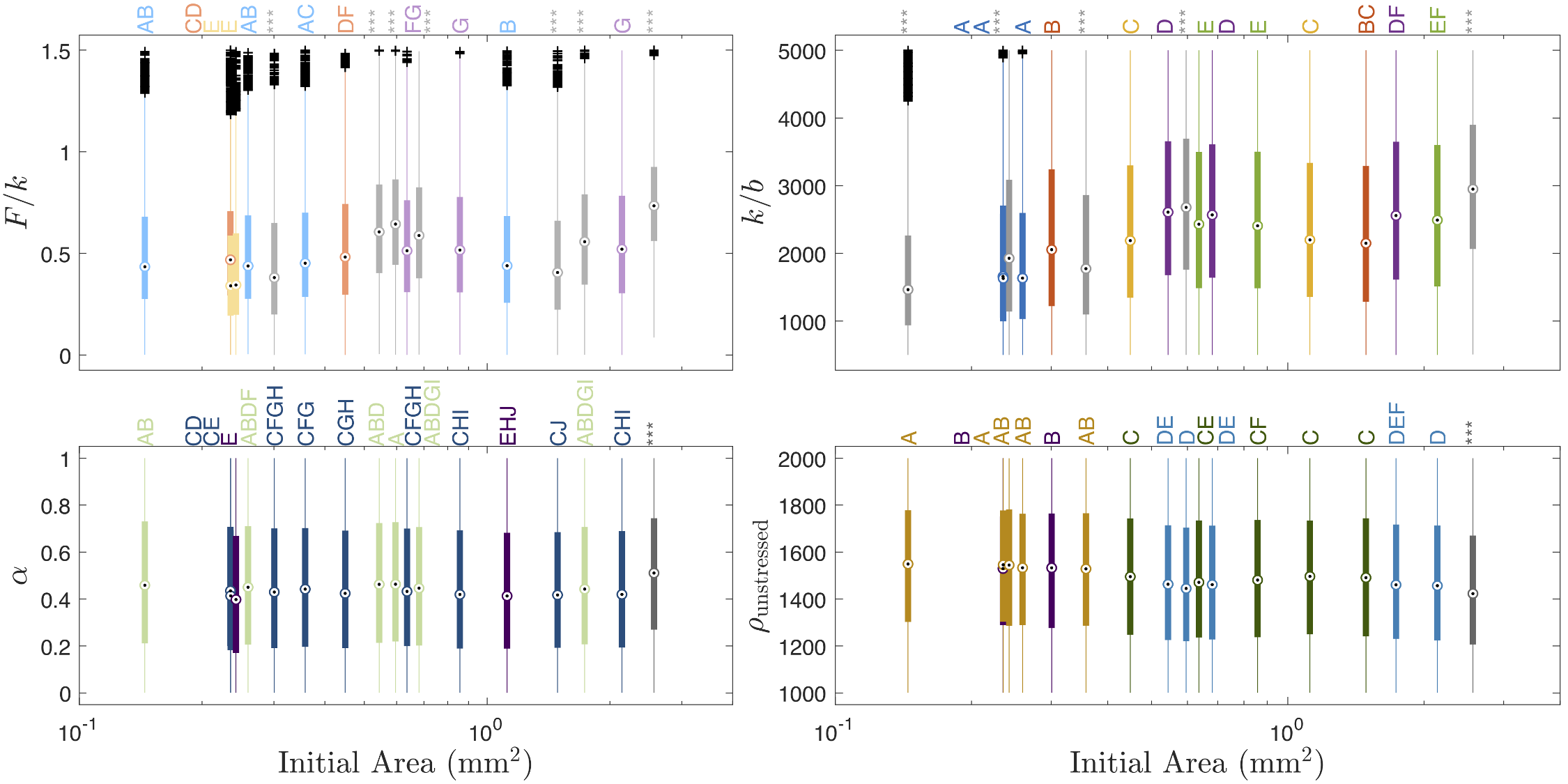

Supplement: S2 Fig — The compact letter display (CLD) and colors reflect the results for Tukey’s multiple comparisons test; in particular, boxplots labeled with the same letter were considered not statistically different (based on significance level 0.05). Boxplots labeled with *** were statistically significantly different than all the other boxplots. Coloring was chosen to reflect groups of boxplots that were labeled similarly. The plus signs indicate outliers, the target symbols indicate the median, and the length of the boxplot indicates the interquartile range. 10,000 parameter sets were used per explant. Cf. Fig 3 for the grouped boxplots. (TIFF) [file pone.0218021.s005.tiff]

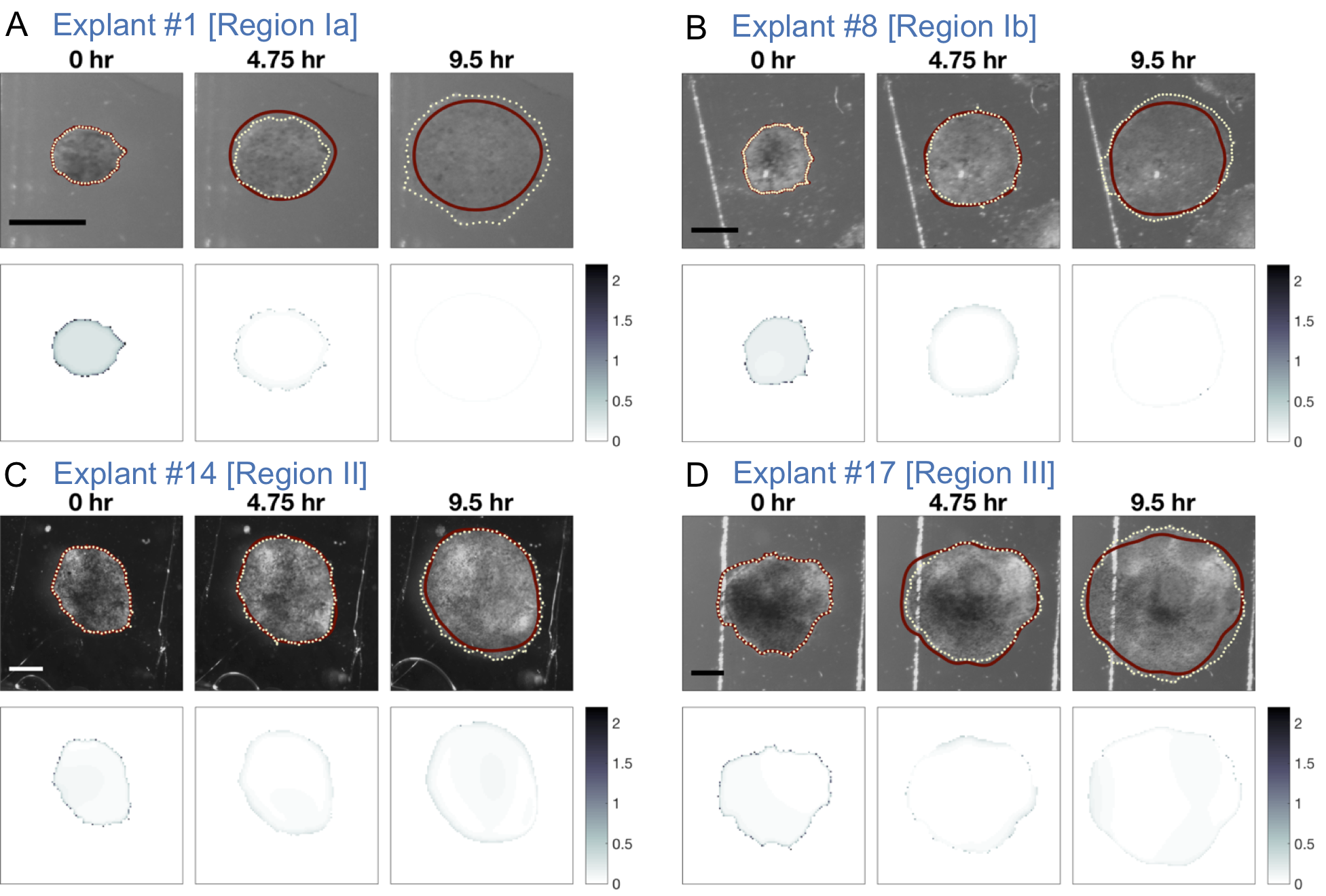

Supplement: S3 Fig — Progression of Xenopus animal cap explant tissue migration at 95 minute time intervals for Explants #1, 8, 14, and 17 with the parameter set that resulted in the smallest error (Eq 12). In the top panel, the computed edge from the mathematical model is represented by a solid dark red curve and the experimental edge is represented by a dotted light yellow curve. The bottom panel shows the absolute value of the density ratio at each grid node (where both density ratios are nonzero) of the experimental data between the given time point and 25 minutes later minus the computed density ratio. Scale bar: 500 μm for each explant. See the S9–12 Videos for time-lapse sequences of these still images. (A) Explant #1 [Region Ia] (initial area 0.14 mm2): F/k = 0.6101, k/b = 510 μm2/h, α = 0.9479 h-1, ρunstressed = 1404 cells/μm2. The total error with these parameters is 1122. (B) Explant #8 [Region Ib] (initial area 0.45 mm2): F/k = 0.9059, k/b = 510 μm2/h, α = 0.9492 h-1, ρunstressed = 1313 cells/μm2. The total error with these parameters is 820. (C) Explant #14 [Region II] (initial area 1.12 mm2): F/k = 0.7948, k/b = 635 μm2/h, α = 0.9410 h-1, ρunstressed = 1530 cells/μm2. The total error with these parameters is 774. (D) Explant #17 [Region III] (initial area 2.14 mm2): F/k = 0.8295, k/b = 950 μm2/h, α = 0.8963h-1, ρunstressed = 1752 cells/μm2. The total error with these parameters is 834. (TIFF) [file pone.0218021.s006.tiff]

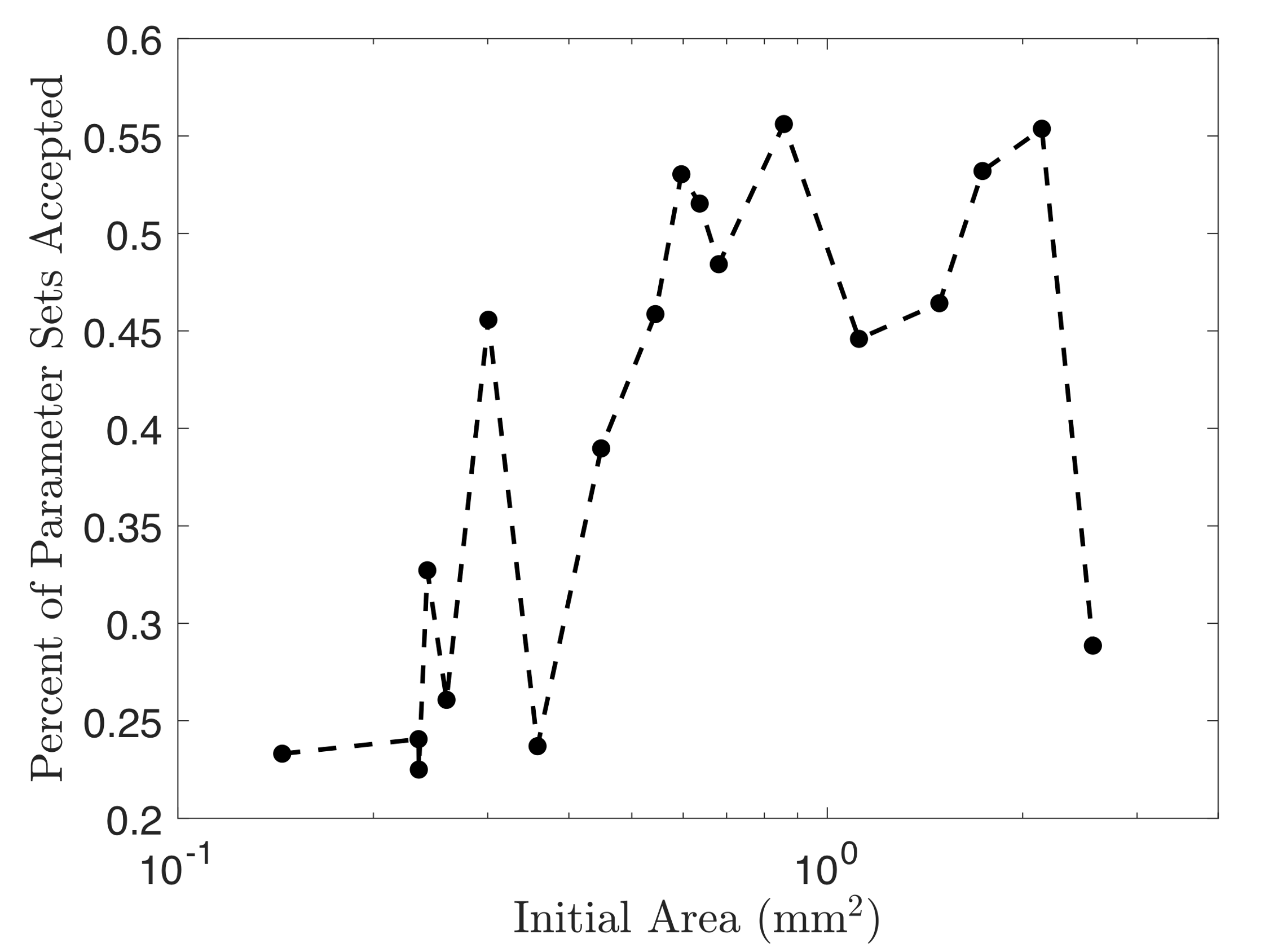

Supplement: S4 Fig — 10,000 parameter sets that resulted in total error (Eq 12) less than or equal to a tolerance threshold of 1500 were collected for each of the 18 explants in the model building set. More than 10,000 simulations were run to obtain 10,000 accepted parameter sets, and in general, smaller explants required more simulations run than larger explants. (TIFF) [file pone.0218021.s007.tiff]

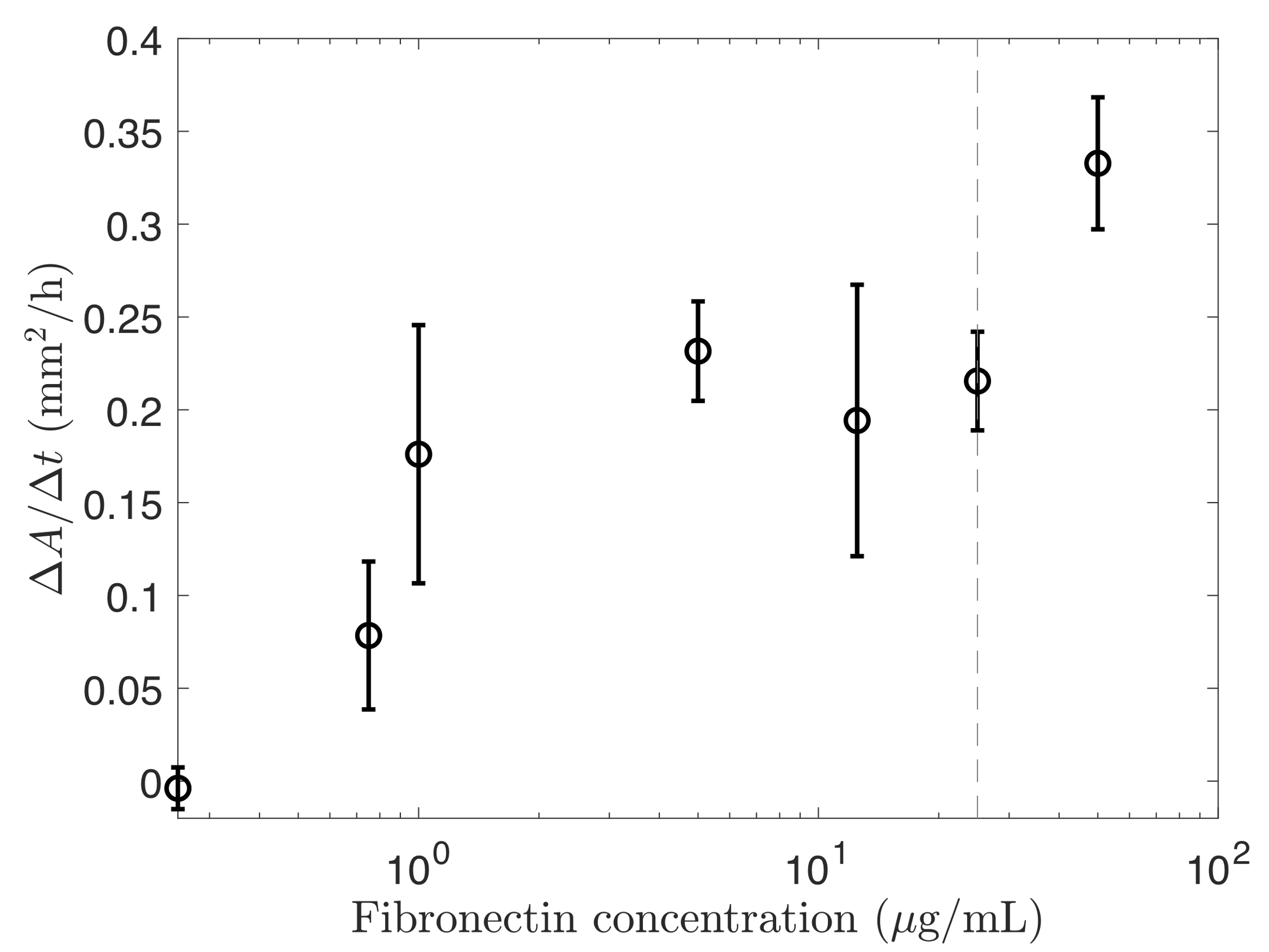

Supplement: S5 Fig — Average change in area over time (ΔA/Δt) for Xenopus animal cap explants that are plated on petri dishes with different concentrations of fibronectin. The error bars show the standard deviation. The spreading rate is faster for higher concentrations of fibronectin than for smaller concentrations of fibronectin. The dashed line represents the fibronectin concentration for the experiments in this paper, 25 μg/mL. (TIFF) [file pone.0218021.s008.tiff]
